# Hypospadias

**DOI:** 10.1155/2008/650135

**Published:** 2008-10-30

**Authors:** N. Djakovic, J. Nyarangi-Dix, A. Özturk, M. Hohenfellner

**Affiliations:** Department of Urology, University of Heidelberg, Medical Center, 69120, Heidelberg, Germany

## Abstract

*Objective*. The great possibility of variations in the clinical presentation of hypospadia, makes its therapy challenging. This has led to the development of a number of techniques for hypospadia repair. This article assesses past and present concepts and operative techniques with the aim of broadening our understanding of this malformation. *Materials and Methods*. The article not only reviews hypospadia in general with its development and clinical presentation as well as historical and current concepts in hypospadiologie on the basis of available literature, but it is also based on our own clinical experience in the repair of this malformation. *Results and Conclusion*. The fact that there are great variations in the presentation and extent of malformations existent makes every hypospadia individual and a proposal of a universal comprehensive algorithm for hypospadia repair difficult. The Snodgrass technique has found wide popularity for the repair of distal hypospadias. As far as proximal hypospadias are concerned, their repair is more challenging because it not only involves urethroplasty, but can also, in some cases, fulfil the dimensions of a complex genital reconstruction. Due to the development of modern operating materials and an improvement in current surgical techniques, there has been a significant decrease in the complication rates. Nonetheless, there still is room and, therefore, need for further improvement in this field.

## 1. INTRODUCTION

### 1.1. Definition, Incidence and Classification

With an incidence of 1:300, hypospadia is one of the most common genital anomalies in male newborns [1]. Hypospadia is defined as an anomaly (hypo- or dysplasia) involving the ventral aspect of the penis [2]. These malformations mainly comprise of an abnormal ventral opening of the urethral meatus, an abnormal ventral curvature of the penis (chordee) and/or an abnormal distribution of the foreskin. The extent of the malformation varies.

Such ectopic urethral openings (meatus) can be located at the tip of the glans penis (hypospadia sine hypospadia), glanular, coronal, subcoronal, along the penile shaft, penoscrotal, scrotal, or perineal. The form and extent of malformation of the urethral opening varies as well and is in some cases widely gaping and resembling the mouth of a fish. A stenosis is rather rare.

Generally, severe forms of hypospadia are typically accompanied by an abnormal ventral curvature of the penis (chordee). This is due to the difference in length between the ventral and the dorsal side of the penis (corporocavernosal dysproportion). Proximal hypospadias frequently have a penoscrotal transposition and/or bifid scrotum.

Further abnormalities in hypospadia concern the prepuce. Typically, there is a dorsal hump with excessive skin on the dorsal and a scarcity of foreskin on the ventral aspect of the penis. In most cases, the frenulum is entirely missing. In the rare cases when the prepuce is normally developed, it must be preserved and a circumcision avoided [3].

### 1.2. Background

The hypospadic penis is often anatomically similar to the normal penis, at least as far as the dorsal aspect is concerned. However, the ventral aspect is pathological: the development of the prepuce incomplete, the formation of the urethral plate into a urethra defective and the corpus spongiosum deficient. Histologically, the urethral plate consists of well-vascularised tissue with large endothelial sinuses lined around an abortive urethral spongiosum. Fibrosis and cicatrisation are rarely available [4]. These characteristics make the urethral plate ideal for urethroplasty [5].

The development of the urethral plate is genetically influenced by cell differentiation, hormonal and enzymatic activity, as well as tissue transformation. Before the 7th week of gestation, the structure of the genital is indifferent [2, 6]. Afterwards, the tissue differentiation, including the elongation of the phallus, the formation of the penile urethra, and the development of the prepuce [7] are influenced by the presence or absence of androgens and the signals of the SRY-gene. More recent studies support the theory of endodermal differentiation. According to this theory, the entire urethra stems from the urogenital sinus [8]. The continual development of the urethral plate into the genital tubercle is followed by the ventral fusion of the urethral folds [9]. The development of the prepuce not only occurs simultaneous to the fusion of the urethral plate, this development in fact depends on it. In cases where the fusion of the urethral plate is altered, the prepuce on the ventral aspect of the penis remains underdeveloped.

Interferences in the androgen metabolism, for example, 5*α*-Reductase deficit, defects of the androgen receptor, or gene defects are possible aetiological factors for hypospadia, that are only found in <5% of the patients [10, 11]. Hypospadia is also found as a part of different syndromes.

The incidence of hypospadia is increasing worldwide. A possible explanation for this trend could be the increasing environmental pollution. In this context, it is known that human beings increasingly ingest substances with estrogen, for example, as found in certain insecticides, natural herbs, and so forth. Animal models demonstrated that estrogens lead to an alteration or even complete interruption of the development of the penis [12]. The aetiology of the majority of hypospadias though remains unknown.

## 2. TREATMENT

Surgical reconstruction is the only possible therapeutic option for hypospadia [2]. The primary objectives of the reconstruction are to create a vertically slit orthotopic meatus, straighten the penis in case of curvature and establish good cosmetic results that include a conically shaped glans. Other important aspects for the reconstruction are to avoid shortening the penis and optimal skin coverage that excludes the use scrotal skin for coverage of the penis.

The optimal age for correction of hypospadia is between the 6th and the 24th month. Thanks to the possibility of topical application of dihydrotestosterone, it is possible to optimise the size of the penis at this early age of operation [13]. In the majority of cases, the operation can be done in one step. A two-step approach is rarely necessary, for example, in case of insufficiency of the urethral plate or hypoplastic skin as often found in Re-Do Hypospadias [14].

Successful hypospadia surgery incorporates the following steps: straightening of the penis (orthoplasty), reconstruction of the urethra (urethroplasty), the meatus (meatoplasty), the glans (glanuloplasty) and the skin of the penis as well as that of the scrotum whenever necessary.

### 2.1. History

The fact that there are over 250 methods of surgical correction of hypospadias described in the literature indicates that the “hypospadiologists” are still in search of the ideal technique. The statement: “There is nothing new in surgery not previously described” [15] is especially true as far as hypospadia repair is concerned, because many so believed new techniques were, as a matter of fact, originally described in historical documents and books.

In the 19th century, Dieffenbach [16] tried to reconstruct a neourethra through secondary epithelialisation by perforating the glans with a canula and therefore establishing a connection to the hypospadic urethra. Theofil Anger first used tubularised local flaps in the 19th century for hypospadia repair [17]. In 1875, Wood introduced the “meatal-based-flap-technique” that is the basic idea behind the Mathieu technique [18]. The idea of reconstructing the neourethra out of a vascularised island flap was also first described in the 19th century. In connection with this, Van Hook described the reconstruction of the neourethra with a dorsal preputial flap. The idea of using a free flap for urethroplasty is also not new. Towards the end of the 19th century, Nove-Josserand used skin grafts for the urethral reconstruction [19].

The above-mentioned techniques were further pursued and advanced in the 20th century. Ombrédanne created the neourethra out of a round local submeatal flap and covered it with a dorsal preputial flap [19]. Similar strategies were followed by Mathieu and Brown. Horton and Devine introduced the so called “Flip-Flap technique” for the correction of distal hypospadias that is favoured by certain surgeons up to date [20]. At the same time, techniques that preferentially use vascularised island flaps were also further pursued. The best known of these is undoubtedly the urethroplasty using a transverse preputial island flap as popularised by Duckett in 1980 [21]. Although several trials were performed in the 20th century with free flaps, buccal mucosa is the only regularly used graft at the moment [22].

The mobilisation and elongation of the urethra is an interesting concept, which can in some cases be used to avoid urethroplasty. Duckett's principle, which is also known as “MAGPI,” is based on this concept [23]. This idea too is not new and was first described by Beck in 1889 [24].

Even though under different conditions, the same concepts are still applied up to date. The surgical results have been further improved by the use of modern sutures, loupe magnification, modern dressing material, better foley catheters, and better methods for the diagnostic and correction of penis curvature through artificial or medically induced erections.

### 2.2. Urethroplasty

The selection of the surgical technique for the reconstruction of the neourethra should not only be influenced by the position of the meatus but must in fact take the entire complex of anomalies—that is, the quality of the urethral plate as well as that of the penile skin, the form of the glans, the length of the urethra, and the grade of corporocavernosal dysproportion—into consideration.

The major techniques of urethroplasty are described in detail under the subtitles “proximal hypospadias” and “distal hypospadias.”

### 2.3. Orthoplasty and Penile Skin Coverage

The penile curvature results from the dysplasia/hypoplasia on the ventral aspect of the penis. Mild curvatures can already be corrected by complete mobilisation of the penile skin. This way, the so-called, “skin chordee” can be released. If the curvature is still existent after such mobilisation, other methods must be applied [25].

The presence of altered fibrotic tissue around the urethral plate and on the ventral aspect of the penis is seldom. This is, therefore, a rather rare reason for penile curvatures. It makes it also easy to understand that a chordectomy alone rarely leads to a straightening of the penis [2]. About 5% of the patients have a so-called corporocavernosal disproportion, which is an effect of the disparity in the development of the tunica albuginea on the ventral and dorsal aspects of the penis [25, 26].

Depending on the extent of the penile curvature, the reconstruction can either be performed per dorsal corporoplasty or with the use of a ventral patch. In the recent past, a number of authors seem to increasingly favour the midline dorsal plication [2]. This technique is based on studies on fetal phallus, which detected that there are no nerves running in the neurovascular bundles in the midline [26]. Mild and moderate curvatures can be corrected with this technique. We correct curvatures up to 40° using Yachia's technique [27]. To avoid extreme shortening of the penis during correction of more severe penile curvatures, a ventral patch, in most cases out of preputial skin, can be used.

The reconstruction of the penile skin is particularly challenging after degloving, “excavation” of the penis, and urethroplasty. In such cases, it is important to pay attention not to embed the penis in the scrotum or the mons pubis. In cases of simultaneous penoscrotal transposition, the anomaly can also be corrected within the same session. This is done with scrotal rotational flaps that are only mobilised up to the subcutaneous layer in order not to compromise the vascularisation of the island flaps and the penile skin [14].

The straightening of the penis as well as an optimal plastic reconstruction of the scrotum and penile skin demands great expertise.

### 2.4. Distal Hypospadias

The majority of patients with this type of hypospadia can urinate with a straight urine stream and have not pronounced penile curvatures. Nonetheless, most parents wish for a “normal penis” for their child. Therefore, the surgical reconstruction of distal hypospadias must meet these cosmetic requirements [2]. The psychosocial aspect is another important factor to consider while making the decision on performing the operative reconstruction in this group of patients.

As of today, the meatal advancement urethroplasty (MAGPI), glans approximation procedure (GAP), Mathieu's procedure and the tubularised incised plate urethroplasty (Snodgrass technique) are among the most established and reliable methods [2].

With the use of the urethral plate for urethroplasty, the complication rate has been clearly reduced. At the beginning, primary tubularisation, also known as the Thiersch-Duplay, was performed in patients with wide urethral plates and deep fossa navicularis [28]. In cases where the urethral plate is narrow, the Mathieu or MAGPI technique or variants thereof are applied. With these methods, a submeatal-based flap is augmented on the narrow urethral plate and the meatus repositioned on the glans [2].

Lately, the concept of incision and tubularisation of the urethral plate with consecutive secondary healing, as popularised by Snodgrass et al. [29], has revolutionised hypospadia surgery [5, 29]. Its low complication rates, excellent cosmetic results and the simple surgical technique have made it very popular among hypospadia surgeons [30]. The initial concerns and, subsequently, reports about increased stenosis have become quite seldom [31, 32]. As long as there is no penile curvature, this technique is the method of choice for distal hypospadias [32]. This method is increasingly applied for the repair of penile hypospadias as well [29].

Nonetheless, there are still complications mostly fistulas reported in [32]. In order to prevent fistulas, particularly healthy tissue from different areas is used to cover the neourethra using different techniques. Retik described the use of asymmetrical flaps from the dorsal penile skin and the prepuce [33]. Other authors use distal extensions of the parting corpus spongiosum to cover the neourethra [34]. Sozubir and Snodgrass used a dorsal dartos flap that was transposed to the ventral aspect of the penis over a buttonhole technique.

**Figure 1:**
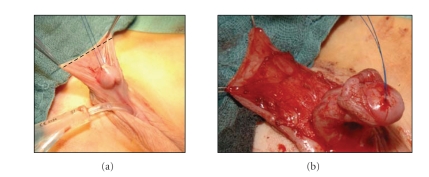
Preparation of the dartos flap.

We, on our part, favour the longitudinal dorsal dartos flap. Mobilising the penile skin involves complete preservation of the dorsal hump. This skin is stretched by two stay sutures, and then incised proximal to the subcoronar region (see Figure 1). The preparation and deepithelialisation of the dartos flap begins proximal to the dorsal hump in an area where the subcutaneous tissue is not pathologically altered. This way, the preparation of the dartos flap is eased and a complete preservation of a well-vascularised dartos flap is possible (see Figure 1).

**Figure 2:**
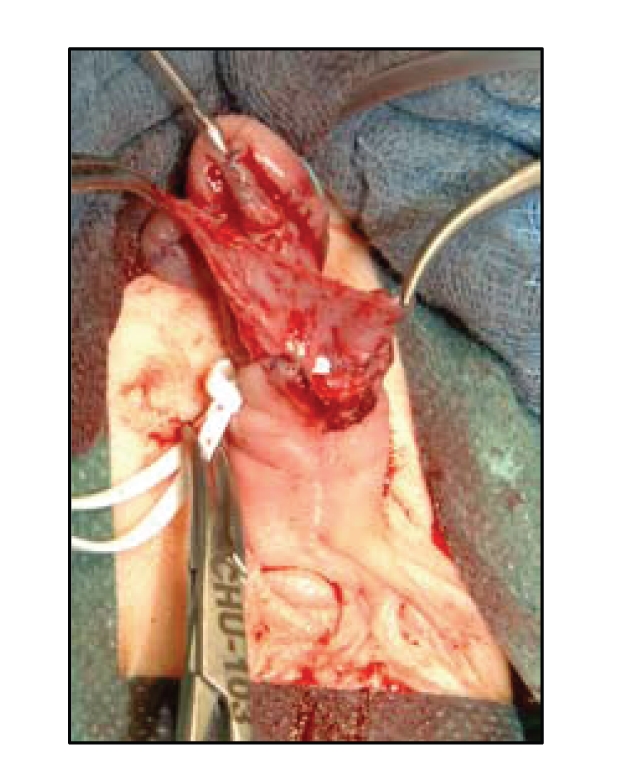
Incised and tubularised urethral plate with the ventrally transpositioned dartos flap.

The urethral plate is mobilised in the layer of Buck's fascia and the tip of the glans incised at the fossa navicularis. In order to enable a tension-free suturing-up of the glans, the incision must be made all the way to the cavernous bodies. After making a midline incision into the urethral plate, it is tubularised with a continuous 7/0 Vicryl suture around a 6 Fr catheter. The dartos flap is then transpositioned to the ventral aspect of the penis with the buttonhole technique and sutured into the incised glans (see Figure 2). This way the neourethra is well covered by this dartos flap.

The glanuloplasty is done with two-layer sutures of Vicryl 7/0. In order to avoid stenosis, it is important to create a wide-enough meatus and evert it afterwards [32].

### 2.5. Proximal Hypospadias

Usually, the intensity of the ventral dys- and hypoplasia increases with increasing grade of hypospadia. That means that the skin on the ventral aspect of the penis and the usability of the urethral plate decreases with an increase in grade of dysplasia. At the same time the penile curvature increases, which makes it sometimes necessary to transect the urethral plate. This special anatomic constitution demands the selection of a surgical technique that is complex and challenging [25].

In such forms of hypospadia, the penis must be first straightened, the urethral plate mobilised up to healthy corpus spongiosum and then the urethroplasty performed with additional tissue. Principally, pedicled or free flaps are used to reconstruct the neourethra in onlay technique. The key to success, in this case, is the preservation of the urethral plate that builds the ventral portion of the neourethra. Most surgeons reconstruct the dorsal portion out of a pedicled inner preputial skin graft [2, 35, 36]. With the integration of the urethral plate, complications like proximal stenosis can be avoided. Furthermore, the fistula rate can be decreased down to 5–10% by using well-vascularised pedicled island grafts [36, 37]. This method has proved its worth in the long run [37–40].

**Figure 3:**
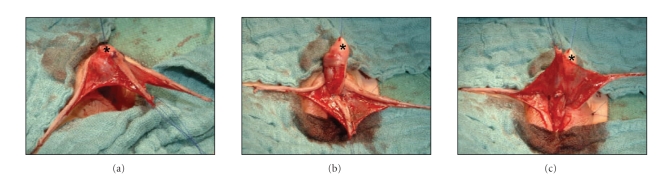
Ventral transposition of the longitudinal dorsal dartos flap with the buttonhole technique. Star shows the glans.

**Figure 4:**
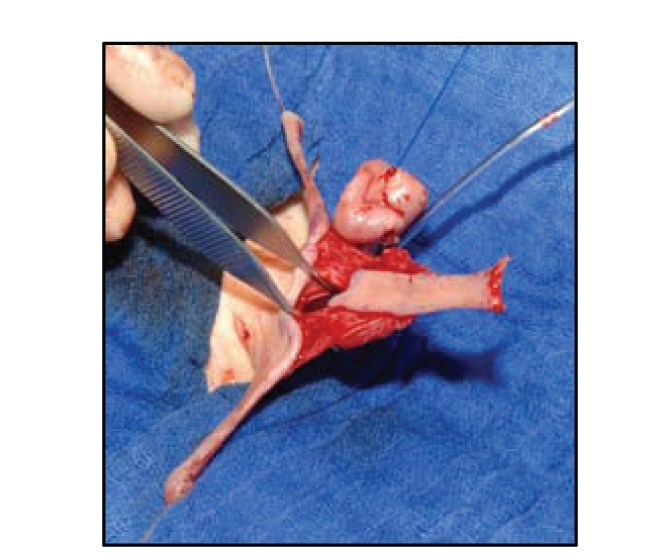
Dorsal perspective of the longitudinal island flap. The graft is right in the centre of the vascular pedicle. The buttonhole is already made.

We generally prefer the use of the longitudinal preputial/penile island flap from the preputial and/or the dorsal penile skin. During preparation of the flap, two lateral devascularised skin portions are developed with the vascularisation in favour of a centrally situated island flap (Figure 3). The devascularised skin is later trimmed off during the reconstruction of the penile skin. The length, width, and shape of this dorsal island flap are formed according to the morphology and quality of the urethral plate. The island flap is transposed ventrally with the buttonhole technique (Figures 3 and 4). Its pedicle contains, in respect to the flap, axially aligned vascularisation.

The reconstruction of the neourethra is done in “onlay technique.” First, the island flap is distally fixed to the hypospadic meatus with interrupted sutures (Vicryl 7/0). Both of the sides of the anastomosis of the onlay are sutured with running suture. Both suture lines of this anastomosis are completely covered by the vascular pedicle of the dorsal island flap. The advantage of this flap over the Duckett-flap is that the longitudinal dorsal island flap lies right in the middle of a wide and well-vascularised pedicle (Figure 4). It is thus an island flap that lies in line with its vascularisation [16, 17]. On the other hand all suture lines are fully covered up by well-vascularised tissue. This way badly perfused borders and corners that are predispose to fistulation are avoided.

**Figure 5:**
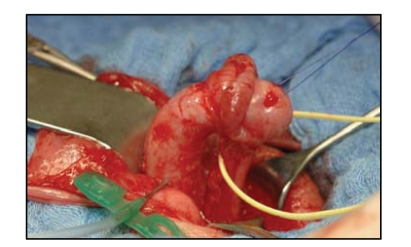
After complete mobilisation of the urethra and artificial erection of the penis, the ventral curvature with a short urethral plate becomes evident.

Generally, the urethral plate can be preserved while straightening the penis. In certain cases though, it must be lifted and incised (Figure 5). In yet other cases, the urethral plate is missing completely. In such patients, a tubularised urethroplasty is performed by using an island flap formed into a role to bridge the missing section of the urethral plate. Due to its increased complication rate, this tubularised urethroplasty has been abandoned by most surgeons. Such complications include segmental strictures and diverticula that occur in up to 69% of the cases [41].

An alternative to tubularised urethroplasty for complex and secondary hypospadias are two-step approaches, the most popular of them being the two-step technique of repair according to Bracka [42, 43]. In the 1st step, the penis is straightened and the cicatrisation of the urethral plate eliminated. A mucosal graft out of the cheek or lower lip is harvested and placed on the prepared bed. The tips of the glans are also reconstructed and lined with the mucosal graft. In the 2nd step, approximately. 6 months later, the mucosal grafts are mobilised, trimmed around the glans and tubularised to a neourethra. Bracka has reported good results in complex hypospadias with this method. Nonetheless, a two-step technique has a relevant disadvantage, namely that of the second operation with its additional complications as well as the negative psychosocial effects on the patient and his family [44].

In order to be able to perform a one-step procedure, a number of studies have been presented in the recent past describing the use of a combination of mucosal grafts and local flaps. In such cases, the defect that results from incision of the urethral plate is bridged over using a mucosal inlay graft and the neourethra is reconstructed out of an island flap in onlay technique and all that in a single procedure [44]. This way, the advantages of a one-step procedure as well as those of the onlay technique are both exploited.

We on our part favour the longitudinal preputial and penile skin island flap. First the penile skin is mobilised, then the urethral plate is incised vertically beginning at the hypospadic meatus all the way to the glans thereby building two glanular wings proximally. This mobilised urethral plate then lies as a groove between both glanular wings. In order to avoid bleeding out of dysplastic lateral branches of the corpus spongiosum, the preparation of the distal section of the urethral plate is done along Buck's fascia.

**Figure 6:**
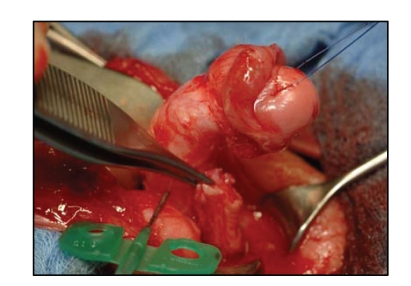
After horizontal incision of the urethral plate the penis is still ventrally curved and a large defect in the distal part of the urethral plate is revealed.

**Figure 7:**
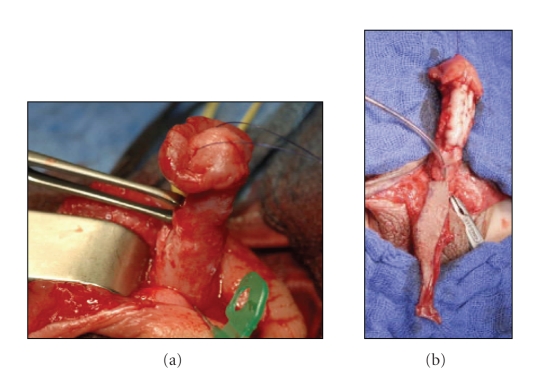
Straightening of the penis (a). The mucosal inlay graft is fixed on the cavernous bodies (b). The longitudinal island flap is ready for the onlay anastomosis (b).

The decision of whether or not to horizontally incise the urethral plate is made depending on the intraoperative findings after artificial erection (Figure 5). We do the incision of the urethral plate proximal to the balanopenile furrow. This way, the proximal stump of the urethral plate is retracted and it, therefore, interrupted/missing along the mid section of the penis (Figure 6). In the next step we straighten the penis and then harvest the buccal mucosal graft. This graft is then perforated to enable drainage of haematoma between the cavernous bodies and the placed graft. We then place the buccal mucosal inlay graft on the cavernous bodies and fix it between the retracted proximal and the distal ends of the urethral plate (Figure 7). In order to enable a large surface of adhesion between the graft and the tunica albuginea, the graft is quilted on to its bed with Vicryl rapid 7/0.

In the same session, a longitudinal preputial/penile island flap won out of the preputial and dorsal penile skin is used for the reconstruction of the neourethra in onlay technique (Figure 7, [35]). Rotational and additionally island skin flaps are used in combination for coverage of the penis. A single-step procedure can be performed in 75% of the children with penoscrotal transpositions [14].

## 3. CONCLUSION

Hypospadia surgery is challenging. The fact that there are wide variations in the presentation and extent of malformations as well as tissue characteristics existent makes every hypospadia individual and a proposal of a universal comprehensive algorithm for hypospadia repair difficult. The Snodgrass technique has found wide popularity for the repair of distal hypospadias. As far as proximal hypospadias are concerned, their repair is complex and could in fact be seen as a form of genital reconstruction. This repair not only involves urethroplasty, but also has its goal in achieving good cosmetic results with a straight normal-proportioned penis and an orthotopic meatus in addition to the functional urethra. Even though the complication rates have decreased, thanks to modern operating materials and an improvement of current surgical techniques, there still is room and therefore need for further improvement in this field.
